# Aberrant Upregulation of RUNX3 Activates Developmental Genes to Drive Metastasis in Gastric Cancer

**DOI:** 10.1158/2767-9764.CRC-22-0165

**Published:** 2024-02-02

**Authors:** Kazuto Suda, Atsushi Okabe, Junichi Matsuo, Linda Shyue Huey Chuang, Ying Li, Nawaphat Jangphattananont, Naing Naing Mon, Khine Nyein Myint, Akihiro Yamamura, Jimmy Bok-Yan So, Dominic Chih-Cheng Voon, Henry Yang, Khay Guan Yeoh, Atsushi Kaneda, Yoshiaki Ito

**Affiliations:** 1 Cancer Science Institute of Singapore, National University of Singapore, Singapore.; 2 Department of Molecular Oncology, Graduate School of Medicine, Chiba University, Chiba, Japan.; 3 Department of Surgery, Yong Loo Lin School of Medicine, National University of Singapore, Singapore.; 4 Innovative Cancer Model Research Unit, Institute for Frontier Science Initiative, Kanazawa University, Japan.; 5 Department of Medicine, Yong Loo Lin School of Medicine, National University of Singapore, Singapore.; 6 Department of Gastroenterology and Hepatology, National University Health System, Singapore.

## Abstract

Gastric cancer metastasis is a major cause of mortality worldwide. Inhibition of RUNX3 in gastric cancer cell lines reduced migration, invasion, and anchorage-independent growth *in vitro*. Following splenic inoculation, CRISPR-mediated RUNX3-knockout HGC-27 cells show suppression of xenograft growth and liver metastasis. We interrogated the potential of RUNX3 as a metastasis driver in gastric cancer by profiling its target genes. Transcriptomic analysis revealed strong involvement of RUNX3 in the regulation of multiple developmental pathways, consistent with the notion that Runt domain transcription factor (RUNX) family genes are master regulators of development. RUNX3 promoted “cell migration” and “extracellular matrix” programs, which are necessary for metastasis. Of note, we found pro-metastatic genes *WNT5A, CD44*, and *VIM* among the top differentially expressed genes in RUNX3 knockout versus control cells. Chromatin immunoprecipitation sequencing and HiChIP analyses revealed that RUNX3 bound to the enhancers and promoters of these genes, suggesting that they are under direct transcriptional control by RUNX3. We show that RUNX3 promoted metastasis in part through its upregulation of *WNT5A* to promote migration, invasion, and anchorage-independent growth in various malignancies. Our study therefore reveals the RUNX3-WNT5A axis as a key targetable mechanism for gastric cancer metastasis.

**Significance:**

Subversion of RUNX3 developmental gene targets to metastasis program indicates the oncogenic nature of inappropriate RUNX3 regulation in gastric cancer.

## Introduction

To date, gastric cancer is the fifth most frequently diagnosed cancer and the third leading cause of cancer mortality worldwide (1). Despite well-established surgical and systemic treatments, prognosis remains poor for a significant number of patients with gastric cancer, due to distant metastasis-related mortality (2, 3). Because the molecular mechanism of metastasis is not fully understood, identification of metastasis driver genes is required for insights to gastric cancer pathogenesis as well as the design of effective treatment (2, 3).

The three-membered Runt domain transcription factor (RUNX) family encodes master regulators of development in multiple tissue types (4). *RUNX1* and *RUNX2* are well established as regulators of hematopoiesis and osteogenesis, and not surprisingly, strongly implicated in malignancies of the respective tissues (4). Earlier studies have indicated involvement of *RUNX3* in the suppression of solid tumors—heterozygous knockout (KO) of *Runx3* in mouse induces adenomas in lung, mammary gland, and intestine in aged mice (5). The *Runx3*^−^*^/^*^−^ mouse stomach epithelium is highly susceptible to the chemical carcinogen N-methyl-N-nitrosourea (MNU) and readily developed invasive stomach cancer following MNU treatment, compared with wild-type mice (6). Moreover, *Runx3* inactivation in the mouse lung is associated with adenoma formation and reduced latency of oncogenic K-Ras–induced adenocarcinoma formation (7). These observations indicate that *Runx3* exerts strong tumor-suppressive function, particularly at early cancer stages.

Yet, paradoxically, *RUNX3* appears to function as an oncogene in leukemia, basal cell carcinoma, ovarian cancer, head and neck cancer, and pancreatic cancer (8–12). In pancreatic cancer, *Runx3* expression levels were dependent on *Smad4* expression—high Runx3 expression, in the presence of *Smad4* inactivation, *Kras* and *p53* oncogenic mutations, correlated with metastatic potential (13). The mechanism underlying RUNX3 oncogenic function remains unclear.

Forty-five percent to 60% of patients with gastric cancer do not express RUNX3 due to hemizygous deletion and hypermethylation of promoter region (14). As for the remaining population of patients with gastric cancer with high RUNX3 expression, the role of RUNX3 has not been examined. Here, we analyze the role of RUNX3 in gastric cancer cells and establish *WNT5A* as its downstream target in driving metastasis.

## Materials and Methods

### Cell Culture

HGC-27 (RRID:CVCL_1279), LMSU (RRID:CVCL_4849), and HEK293T (RRID:CVCL_0063) were purchased from CellBank Australia, JCRB cell bank, and ATCC, respectively. They were cultured in RPMI1640 Medium (Nacalai tesque), supplemented with 10% FBS (Biowest) and 1% Penicillin-Streptomycin (Gibco by Life Technologies). Cell lines were authenticated by DNA profiling (Promega). All cell lines were tested regularly to be *Mycoplasma*-free by Mycoplasma detection assay (Lonza).

### *Ex Vivo* Cell Culture from Patient-derived Xenograft Tumors

Establishment and maintenance of GAS24 patient-derived xenograft (PDX)-derived cell line were performed as described previously (15). The cultures were maintained in DMEM supplemented with 10% FBS and 1% Penicillin-Streptomycin.

### Reagent and Transfection

The SMARTpool siRNA reagents for RUNX3 (si-RUNX3), WNT5A (si-WNT5A), and nontargeting oligonucleotides were purchased (Dharmacon) as pools of four different sequences (5′-3′): nontargeting control (A)UGGUUUACAUGUCGACUAA, (B)UGGUUUACAUGUUGUGUGA, (C)UGGUUUACAUGUUUUCUGA, (D)UGGUUUACAUGUUUUCCUA; RUNX3 (A) CCUCGGAACUGAACCCAUU, (B) CCUCGGCCGUCAUGAAGAA, (C) GCCGUUCCCUGACCGCUUU, (D) UGACUGUGAUGGCAGGCAA; WNT5A(A)GCCAAGGGCUCCUACGAGA, (B)GUUCAGAUGUCAGAAGUAU, (C)CAUCAAAGAAUGCCAGUAU, (D)GAAACUGUGCCACUUGUAU. Cells were cultured to a confluence of approximately 50% and transfected with 40 nmol/L of siRNA using jet PRIME reagent and jet PRIME buffer (Polyplus transfection). The knockdown (KD) efficiency was determined by qRT-PCR or immunoblot. Transfected cells were harvested 48 hours later for further analysis. For RUNX3 overexpression (OE), we transfected 0.9 µg of pEGFP vector (Clontech) and 0.1 µg of human RUNX3-pEGFP vector (16) into cells. A total of 1 µg of pEGFP vector was included as a negative control. Stable transfectants were generated by antibiotic selection in media containing 400 µg/mL G418. RUNX3 protein expression in stable transfectants were confirmed by Western blot analysis, while *RUNX3* copy number was assessed by qPCR. Recombinant Human/Mouse Wnt-5a protein was purchased from R&D Systems and used at a concentration of 0.1 mg/mL.

### Genome Engineering by CRISPR/Cas9

The single-guide RNA (sgRNA) sequence including PAM region targeting exon 3 of *RUNX3* was determined by the CRISPR Design Tool (http://www.genscript.com/CRISPR-gRNA-constructs.html) as follows: 5′-GGACGTGCCGGATGGTACGG TGG-3′. Gene-specific sgRNA oligos were cloned into a pLenti CRISPR v2 plasmid (Genscript), which bicistronically expresses Cas9 nuclease. HEK293T cells were cocultured with 15 µL of TransIT-LT1 (Mirus Bio) reagent and 5 µg of plasmids comprising sgRNA and packaging vectors PLP1, PLP2, and PLP/VSVG (Addgene). After 48 to 72 hours, virus-containing supernatant was added to the target cells in the presence of 5 µg/mL polybrene (Sigma-Aldrich). After 72 hours, puromycin (Invitrogen) was added for 5 days to select for transduced cells. RUNX3 deletion was confirmed at mRNA and protein levels after single-cell cloning.

### Western Blot Analysis

Cells were lysed with RIPA buffer supplemented with a protease inhibitor cocktail (1:50, Roche) and phosphatase inhibitor cocktail (1:100, Thermo Fisher Scientific). After incubation on ice for 20 minutes, the lysate was centrifuged at 10,000 rpm for 15 minutes at 4°C and the supernatant was collected. Protein was quantitated by the GeneQuant 1300 (GE Healthcare) and loaded at equal amounts to each well of a 4%–20% SDS-PAGE gel. Immunoblots were performed using the following primary antibodies: anti-RUNX3 D6E2 (1:1,000; Cell Signaling Technology), anti-WNT5A 6F2 (1:1,000; LSBio), anti-GAPDH 14C10 (1:1,000; Cell Signaling Technology). Proteins were separated by SDS-PAGE and transferred to polyvinylidene difluoride membranes (Bio-Rad). After blocking in 5% milk in Tris-buffered saline with Tween (TBST) (0.1% Tween-20), the membranes were incubated with primary antibodies. Signals were detected by horseradish peroxidase–conjugated secondary anti-rabbit (1:10,000, GE Healthcare) or mouse antibody (1:10,000, GE Healthcare) and visualized with Image Quant LAS 500 (GE Healthcare).

### qRT-PCR Analysis

RNA was extracted using the RNeasy Mini Kit (QIAGEN), with the use of QIAshredder. cDNA was synthesized using TaqMan reverse transcription reagents kit (Applied Biosystems). qPCR was performed using iTaq Universal SYBR Green Supermix (Bio-Rad) on a QuantStudio 3 PCR system (Thermo Fisher Scientific) according to the manufacturer's instructions. All samples were run in triplicate. Primers were designed to generate a PCR product of <250 bp as follows: *GAPDH* forward 5′-ACCACAGTCCATGCCATCAC-3′, reverse 5′-TCCACCACCCTGTTGCTGTA-3′; *RUNX3* forward 5′-TGGCAGGCAATGACGAGAAC-3′, reverse 5′-TTCCGAGGTGCCTTGGATTG-3′. Thermal cycling conditions were 95°C for 4 minutes followed by 35 cycles of 60 seconds at 95°C, 60 seconds at 60°C, and 60 seconds at 72°C. Expression levels were normalized to the *GAPDH* housekeeping gene.

### Cell Proliferation Assay

A total of 2.0 × 10^3^ of cells were seeded into a 96-well plate. Proliferation was measured daily using the WST-1 (Sigma-Aldrich) according to the manufacturer's Instructions for 4 days. Absorbance readings were measured with a plate reader at 440 nm.

### Invasion/migration Assay

To measure cell migration activity, Polystyrene plates with 6.5 mm Transwell with 8.0 µm pore polycarbonate membrane insert (Corning) were used. For invasion assay, the surface of upper chamber was additionally coated with 50 µL of Matrigel (Corning). A total of 4 × 10^5^ of cells suspended in 200 µL of RPMI1640 with 0.1% BSA were added to the upper chamber. Lower chambers were filled with medium containing 20% FBS as chemoattractant. After incubation at 37°C for 12 to 48 hours (according to cell type), the cells that migrated/invaded to the lower side of the upper chamber were counted. Nonmigrated cells were removed by swabbing top surface of the membrane insert. Membrane containing invading cells was fixed with methanol, and stained with hematoxylin (3 minutes) and eosin (1 minute), and mounted on slides. The migrated cells were counted under light microscope for 5 fields randomly.

### Colony Formation Assay

For soft agar assay, cells (3.0 × 10^3^ per well) were plated in triplicate in 1 mL of 0.35% agarose over a base layer of 0.75% agarose in 6-well plates. After incubation for 14 days at 37°C, the colonies were stained with 0.01% crystal violet in 2% ethanol. For Matrigel assay, cells (3.0 × 10^3^ in 50 µL of Matrigel) were plated on 24 well-plates. Cells were cultured for 7 days at 37°C. Colonies were photographed under microscope and counted by Image J software.

### Formation of Xenografts, Orthotopic Transplantation Model, and Liver Metastasis by Splenic Inoculation

A total of 1 × 10^6^ cells of HGC-27 control and RUNX3 KO cells were resuspended in 40 µL of Matrigel, followed by subcutaneous implantation, orthotopic transplantation into the serous layer of the recipients’ stomach, and also inoculation into the spleen to induce the liver metastasis of 8 to 10 weeks old NOD SCID gamma mice (NSG mice; The Jackson Laboratory) under general anesthesia by isoflurane. At the end of the experiments (4 weeks after cell injection), the recipient mice were sacrificed and tumors harvested and photographed. Tumors in spleen and liver were subjected to histologic analysis and qRT-PCR. Survival curve was plotted from the day of splenic injection. All animal work was performed according to experimental protocols approved by the Institutional Animal Care and Use Committee.

### RNA Sequencing Analysis

Total RNA was extracted from HGC-27 cells using the QIAGEN RNeasy Mini Kit. RNA quality was determined using the Agilent 2100 Bioanalyzer (Agilent technologies). Samples were sent to Beijing Genomics Institute for transcriptome library preparation and sequencing. Sequenced reads were aligned with the STAR software to hg19, and mapped counts were employed to generate the raw expression counts using the FeatureCounts with GENCODE transcriptome annotation. The raw expression counts were further normalized using the cross-correlation method (17). Gene expression levels were expressed as reads per kilobase of exon per million mapped sequence reads (RPKM). Genes which were differentially expressed in HGC-27 between control and RUNX3 KO cells were obtained according to the following criteria: Fold change ≥2 and *P* value ≤0.05. Normalized gene expression data were subjected to analysis of enriched pathways and enriched gene ontology (GO) terms. They were obtained for the differentially expressed genes using DAVID (Database of Annotation, Visualization and Integrated Discovery) v6.8.

### Chromatin Immunoprecipitation and Library Construction

Chromatin immunoprecipitation (ChIP) assays were conducted from approximately 5 × 10^6^ cells as reported previously (18, 19). Briefly, cells were cross-linked with 1% formaldehyde. Cross-linked chromatin was sonicated to a size of 0.2–1 kb using an ultrasonic disruptor. A total of 2–5 µg of antibody and 20 µL of Protein G sepharose beads or 20 µL of anti-rabbit IgG Dynal magnetic beads were mixed in immunoprecipitation (IP) dilution buffer. After washing with IP dilution buffer, antibody-binding beads were added to the sonicated-chromatin sample and incubated overnight at 4°C. Chromatin was eluted after washing beads, followed by reversal of the cross-linking and DNA purification. Chromatin-immunoprecipitated DNA was dissolved in elution buffer (EB) (Qiagen). Libraries were constructed by using KAPA Hyper Prep Kit (KAPA Biosystems) according to the manufacturer's instructions. Chromatin immunoprecipitation sequencing (ChIP-seq) libraries were quantified by Tapestation (Agilent) and sequenced at a concentration of 4 pmol/L on an Illumina Hiseq or NEXTseq (Illumina). Antibodies for histone and RUNX3 are followed as; Anti H3K4me3 (Active Motif), Anti H3K4me1 (Cell Signaling Technology), Anti-Runx3 (MBL, R3-5G4).

### ChIP-seq Analysis

Sequenced reads in ChIP-seq experiment were mapped to UCSC human genome (hg19) using bowtie. Duplicated reads were removed with Picard tools. Peak calling and motif analysis were performed by using HOMER software (http://homer.ucsd.edu/homer/; RRID:SCR_010881). GO analysis was performed by using Metascape (http://metascape.org/gp/index.html#/main/step1; RRID:SCR_016620). HOMER was also used to get differential peaks. Enhancer annotation to the nearest genes was performed by using GREAT (http://bejerano.stanford.edu/great/public/html/index.php). Peak heat maps were produced with the use of HOMER and TreeView for enrichment calculation and visualization.

### H3K27ac HiChIP

H3K27ac HiChIP libraries for HGC27 cells were prepared as described previously (20). In brief, 15 million cells were cross-linked with fresh 1% formaldehyde for 10 minutes at room temperature. Excess formaldehyde was quenched with 0.125 mol/L glycine for 5 minutes and cells washed three times for 5 minutes in ice-cold PBS. Cross-linked cells were lysed in lysis buffer (10 mmol/L Tris-HCl pH 8.0, 10 mmol/L NaCl, 0.2% Igepal CA-630 and protease inhibitors) on ice for 30 minutes. The lysate was sedimented and the pellet resuspended in CutSmart Buffer (NEB) containing 0.1% SDS, and incubated at 65°C for 10 minutes. SDS was quenched on ice with 1% Triton X-100 and sample digested with MboI overnight at 37°C. Digested fragments were labeled with biotin-14-dATP and blunt-end ligated. Ligated chromatin samples were fragmented using Covaris (Fill Level: 10, Duty Cycle: 5, PIP: 140, Cycles/Burst: 200, Time: 4 minutes). Fragmented chromatin was incubated overnight with anti-H3K27ac antibody at 4°C. Washed protein A Dynabeads was added to the samples and incubated for 2 hours at 4°C. Chromatin was eluted after washing beads, followed by reversal of the crosslinking and DNA purification. Biotinylated DNA fragments were pulled down with streptavidin beads and performed tagmentation with Tn5 transposase (Illumina, catalog no. 20034198). Bead-conjugated DNA was amplified by PCR and libraries were sequenced on an Illumina NextSeq 500.

### H3K27ac HiChIP Analysis

*In situ* Hi-C libraries for HGC27 cells were processed using HiC-Pro (21). Significant interactions of H3K27ac HiChIP were calculated using FitHiChIP in 10-kb and 25-kb resolution (22). The WashU Epigenome Browser (http://epigenomegateway.wustl.edu/) was used for visualization of significant interactions (23).

### Collection of Primary Gastric Cancer Specimen

Resected gastric cancer tissues were collected from patients who underwent gastric cancer resection at National University Hospital with patient consent in the Gastric Cancer Biomarker Discovery II study, and which was approved by the Institutional Review Board – Domain Specific Review Board of National Healthcare Group (ref. no. 2005/00440) following Declaration of Helsinki and the ethical principles in the Belmont Report. All patients had provided written informed consent prior to their participation in the study. Clinical information was collected with the approval of the Institutional Review Board.

### Immunofluorescence Study

Paraffin section slides were deparaffinized, described as above. In brief, sections were pretreated by autoclave at 121°C for 20 minutes in antigen retrieval solution (DAKO) to retrieve antigenicity. Sections were blocked by incubation in 5% skim milk or protein block serum-free (DAKO). Primary antibodies specific for anti-RUNX3 D6E2 (1:250; Cell Signaling Technology) and anti-WNT5A 6F2 (1:400; LSBio) were applied to the slides and incubated at 4°C overnight. The samples were treated with conjugated secondary antibody (Invitrogen). Nuclei were stained with 4′,6-diamidino-2-phenylindole (Sigma). Cells were then analyzed by fluorescence microscopy (Zeiss). RUNX3-expressing lymphocytes were distinguished from epithelial cells by E-cadherin. E-cadherin (1:200; BD Pharmingen) or Ki67 (1:250; Invitrogen) positive cells were screened when we counted RUNX3 positive or WNT5A positive cells in gastric cancer specimens.

### Gene Expression Microarray Analysis

Normalized RNA sequencing (RNA-seq) data produced by The Cancer Genome Atlas (TCGA) were downloaded from cBioportal (www.cbioportal.org, TCGA Provisional; RNA-seq V2) and UCSC Xena (xena.ucsc.edu/). Data were available for over 400 of the gastric cancer samples TCGA subjected to mRNA expression profiling. The relationship between the mRNA expression of RUNX3 and clinical course or progression was conducted. Kaplan–Meier plotter provided survival statistics over 800 of the patients with gastric cancer subjected to mRNA expression profiling kmplot.com/analysis/; RRID:SCR_018753)

### Statistical Analysis

All results are expressed as mean ± SD. Statistical analysis was performed by GraphPad Prism (RRID:SCR_002798). A log-rank test was performed to assess statistical significance between survival curves. All tests were two tailed. *P* value <0.05 was considered statistically significant.

### Data Availability

All data relevant to the study are included in the article or uploaded as Supplementary Data. The RNA-seq, ChIP-seq, HiChIP data are available at NCBI Gene Expression Omnibus under the accession numbers GSE250207, GSE250481, and GSE250480, respectively. Any additional information and data will be available from the corresponding author upon reasonable request.

## Results

### Elevated RUNX3 Expression in Patients with Gastric Cancer is Associated with Poor Prognosis

RUNX3 expression in cancer has been extensively studied (4). Silencing of RUNX3 expression by hypermethylation of the CpG island in the RUNX3 P2 promoter was detected in diverse cancer types, including gastric cancer (4). Although we found that RUNX3 functioned as a tumor suppressor in gastric cancer, it was reported that RUNX3 served as an oncogene in epithelial ovarian cancer, basal cell carcinoma, and head and neck squamous cell carcinoma.

We observed strong RUNX3 expression in metastatic tumors induced by the *Pgc-CreERT2;Kras^G12D/+^*;*Apc^flox/flox^;Trp53^flox/flox^* stomach cancer mouse model (24). Moreover, we noted RUNX3 expression in histopathologic vascular invasion and lymph node metastasis from stomach cancer. TCGA database showed that RUNX3 expression in normal tissues were much lower than that in primary tumors (Supplementary Fig. S1A), and patients at stage IV expressed higher RUNX3 mRNA levels than those of stage I (Supplementary Fig. S1B). RUNX3 mRNA level was higher in relapsed and progressed patients despite systemic treatment than patients with disease-free (Supplementary Fig. S1C). In the Kaplan–Meier plotter database (25), patients with high *RUNX3* expression were linked to poor survival rate (Supplementary Fig. S1D). Collectively, these data indicate that the elevated RUNX3 expression could be associated with invasive and metastatic characteristics.

### RUNX3 Promotes Cancer Cell Migration, Invasion, and Anchorage-independent Growth in Gastric Cancer Cells Lines

**FIGURE 1:**
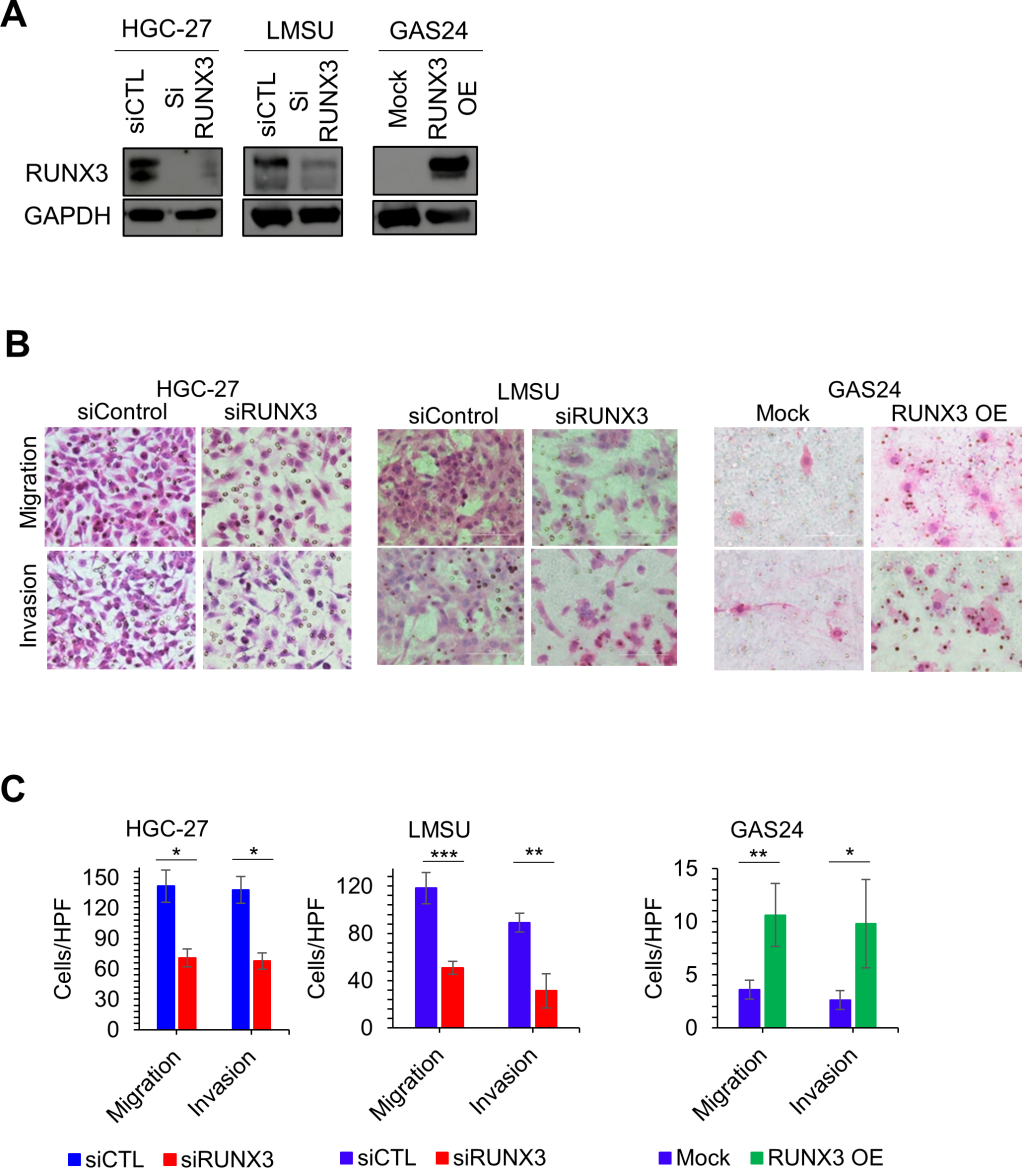
RUNX3 promotes cancer cell migration and invasion. **A,** The efficiency of siRUNX3 KD in HGC-27 and LMSU and RUNX3 OE in GAS24 were validated by immunoblot. **B,** Invasion and migration analysis for siRUNX3 in HGC-27 and LMSU and also RUNX3 OE in GAS24. Experiments were repeated three times. Typical images from one experiment are shown. **C,** Cell invasion and migration were counted and quantified from 5 different high-power fields in each experiment; ***, *P* < 0.001; **, *P* < 0.01; and *, *P* < 0.05 by a two-tailed Student *t* test.

To explore the effects of RUNX3 on metastatic potential, we conducted siRNA-mediated KD of RUNX3 in RUNX3-expressing gastric cancer cell lines, such as HGC-27 and LMSU. As a comparison, we overexpressed RUNX3 (OE) in GAS24, which is a RUNX3-deficient PDX-derived cell line (Fig. 1A). Cell migration and invasion were significantly reduced by RUNX3 KD in HGC-27 and LMSU. RUNX3 OE increased cell migration and invasion in GAS24 (Fig. 1B and C). These data indicate that RUNX3 promotes metastasis-associated functions in gastric cancer cells.

We next used the HGC-27 cell line, a poorly differentiated carcinoma isolated from a metastatic lymph node of a patient with gastric cancer, as a model to study metastasis. A RUNX3-deficient HGC-27 cell line was generated by CRISPR/Cas9 KO (Supplementary Fig. S2A and S2B). A comparison of the growth rates of HGC-27 control and RUNX3 KO using WST-1 assay revealed that HGC-27 RUNX3 KO grew slightly slower than the control (Supplementary Fig. S2A and S2C), thereby indicating that impaired migration/invasion abilities were not due to proliferation differences between control and RUNX3 KO. Migration/invasion assay performed on RUNX3 KO cells confirmed the results obtained by RUNX3 KD (Supplementary Fig. S2D and S2E). Conversely, reintroduction of RUNX3 to RUNX3 KO HGC-27 cells restored cell migration and invasion properties (Supplementary Fig. S2F–S2H). Moreover, soft agar colony formation assay showed that *RUNX3* KO reduced anchorage-independent growth (Supplementary Fig. S2I and S2J).

### Metastasis of Gastric Cancer is Driven by RUNX3

**FIGURE 2:**
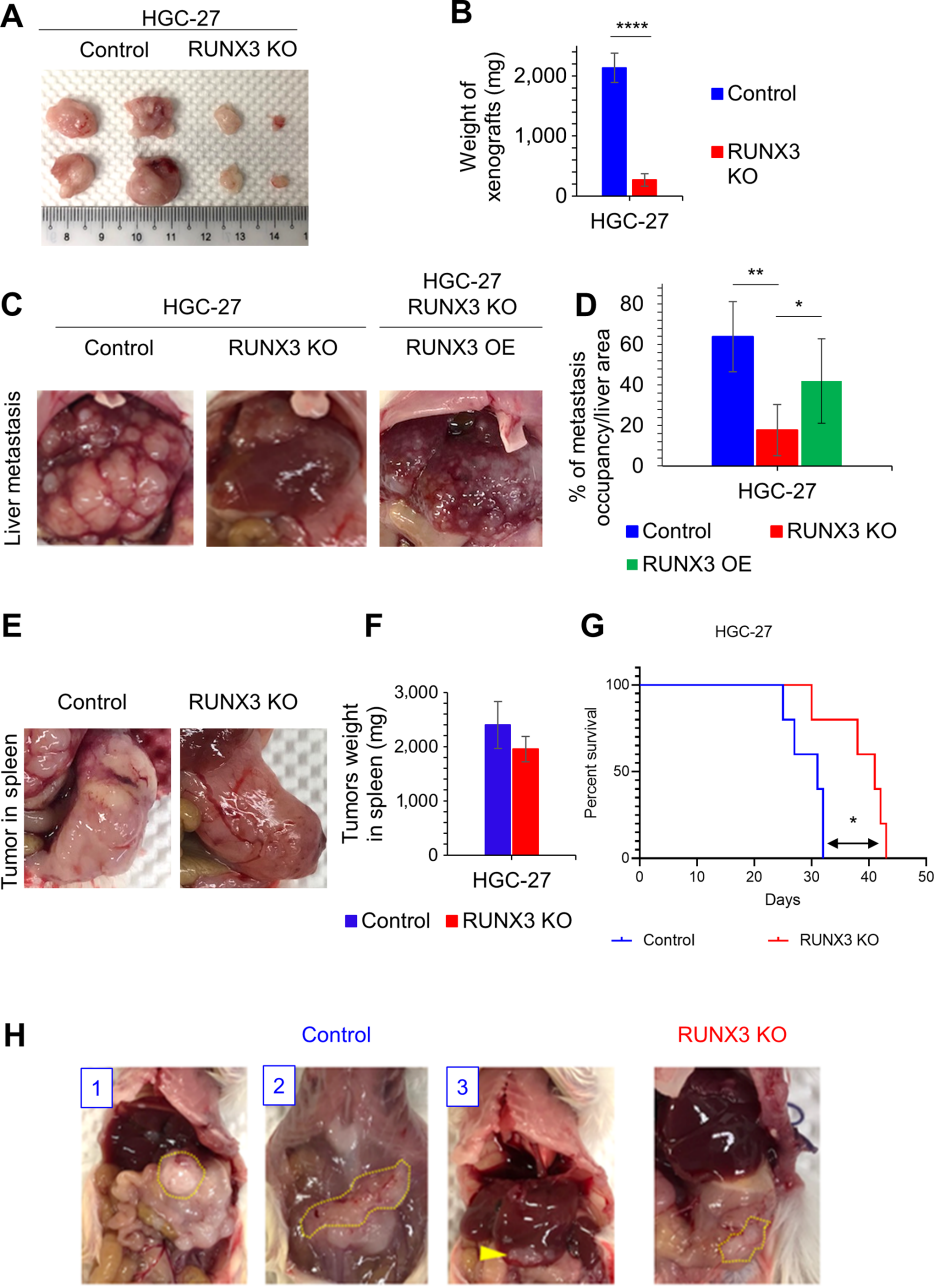
RUNX3 drives metastasis in gastric cancer cells. **A,** Subcutaneous xenograft tumors were obtained by inoculation of 1 × 10^6^ HGC-27 control and RUNX3 KO cells, respectively. Typical images are shown (*n* = 5). **B,** The weight of tumors was quantified; ****, *P* < 0.0001 by a two-tailed Student *t* test. **C,** Liver metastasis model by splenic inoculation of 1 × 10^6^ HGC-27 cells. Representative images of tumors in liver metastasis in control, RUNX3 KO, and RUNX3 KO rescued with reintroduction of RUNX3 (RUNX3 OE) cells are shown (*n* = 5, respectively). **D,** Percentage of the metastatic tumor area in the liver tissue was measured using Image J and graphed (mean + SD); **, *P* < 0.01; *, *P* < 0.05 by a two-tailed Student *t* test. **E,** Representative images of tumor formation in spleen by inoculation of 1 × 10^6^ HGC-27 cells in control and RUNX3 KO are shown (*n* = 5, respectively). **F,** The weight of tumors in spleen was quantified. **G,** Kaplan–Meier plots in liver metastasis models present overall survival for mice after inoculation of control and RUNX3 KO of HGC-27 cells; *, *P* < 0.05 by a two-tailed Student *t* test. **H,** Orthotopic transplantation model of 1 × 10^6^ HGC-27 cells. Representative images of tumors in stomach (1 in blue, circled by dot line), peritoneal invasion (2, circled by dot line), and liver metastasis (3, indicated by an arrow head) in control, and also tumors in stomach (circled by dot line) in RUNX3 KO cells are shown (*n* = 5).

We next examined the *in vivo* activities of RUNX3 in tumor development and metastasis by subcutaneous and splenic inoculation of HGC-27 cells into NSG mice. The growth of subcutaneous xenograft tumors was significantly suppressed by RUNX3 KO (Fig. 2A and B). Furthermore, splenic inoculation of the cells into NSG mice revealed dramatic reduction of liver metastasis for RUNX3 KO cells, when compared with control HGC-27 cells (Fig. 2C and D; *n* = 5). Importantly, reintroduction of RUNX3 to RUNX3 KO HGC-27 cells restored metastatic ability (Fig. 2C and D). In contrast to the reduction of metastasis in RUNX3 KO, the sizes of primary tumors at the splenic inoculation site were similar between control and RUNX3 KO cells (Fig. 2E and F). Therefore, while RUNX3 KO did not significantly affect primary tumor growth, it significantly reduced metastatic outgrowth. The recipient mice with HGC-27 control cells, possibly due to higher metastasis burden, showed shorter survival when compared with RUNX3 KO (Fig. 2G). qPCR and immunofluorescence staining showed significantly higher expression of RUNX3 mRNA and protein in liver metastasis, compared with spleen tumor cells (Supplementary Fig. S3A–S3C), indicating that high RUNX3 expression is associated with metastasis. Orthotopic transplantation model demonstrated that HGC-27 control cells colonized into recipients’ stomach (80%, Fig. 2H, panel 1), directly invaded into peritoneal layer (60%, Fig. 2H, panel 2), and also metastasized to liver (60%, Fig 2H, panel 3), while RUNX3 KO cells only showed localized tumors in the stomach (60%; Fig. 2H, RUNX3 KO). These data suggest that RUNX3 drives aggressive metastasis of gastric cancer cells.

### Transcriptomic Profiling of HGC-27_CONTROL and HGC-27_RUNX3KO Cells Reveal RUNX3-related Transcriptional Program in Driving Metastasis

**FIGURE 3:**
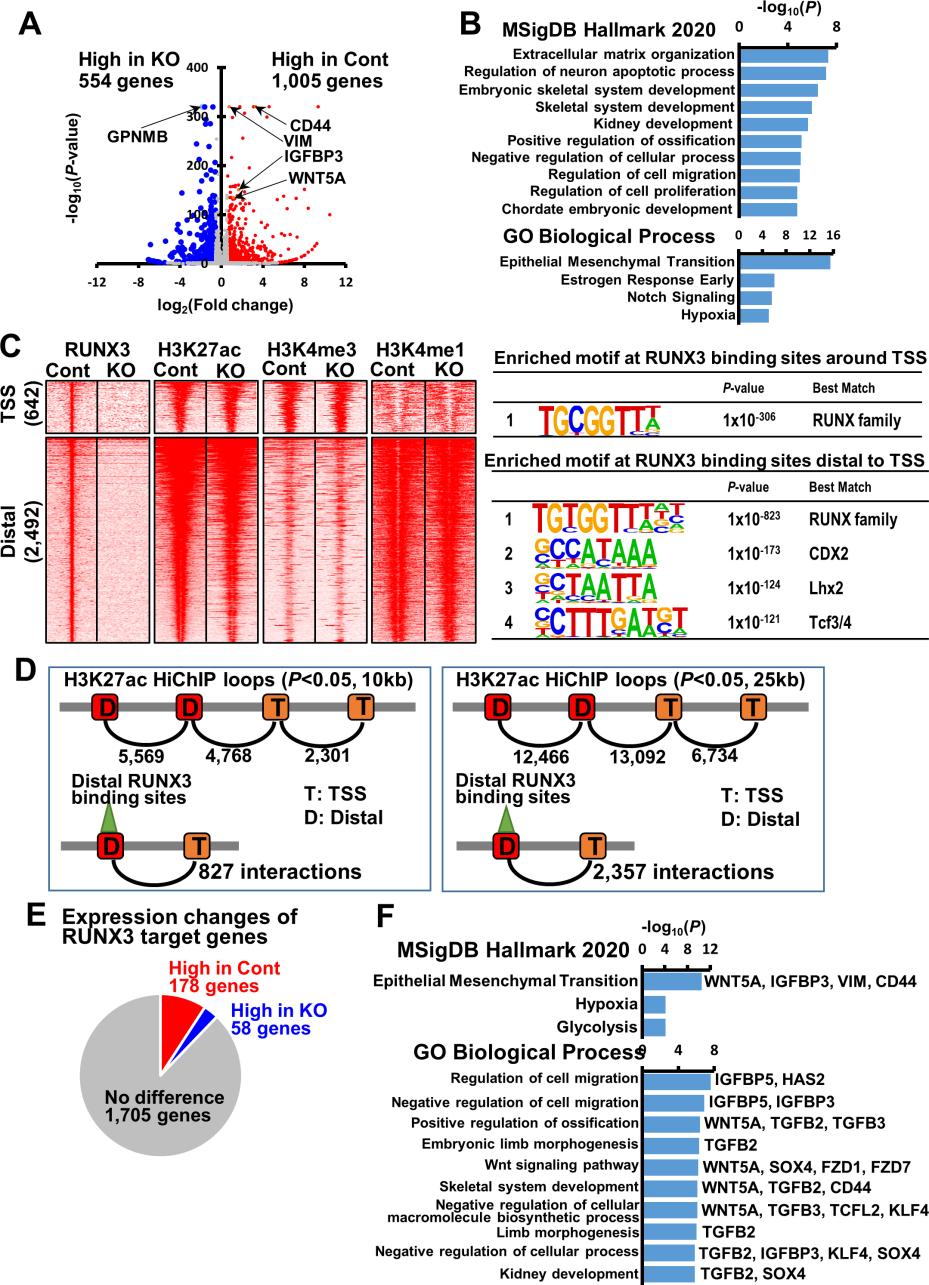
RUNX3 drives a metastatic transcriptional program. **A,** A volcano plot obtained from RNA-seq. A total of 1,005 genes significantly downregulated (*P* < 0.05) in RUNX3 KO cells were identified, including CD44, Vimentin, IGFBP3, and WNT5A (marked in red). A total of 554 genes significantly upregulated (*P* < 0.05) in RUNX3 KO cells were identified, including GPNMB (marked in blue). All dots are visualized on the basis of expression alteration by ≥2-fold change by RUNX3 KO. **B,** Analysis of enriched pathway obtained from MSIgDB Hallmark 2020 and Gene Ontology Biological Process of significantly downregulated genes in RUNX3 KO cells (*n* = 1,005). **C,** Heat maps representing read densities of RUNX3, H3K27ac, H3K4me3 (promoter mark), and H3K4me1 (enhancer mark) in ±5-kb regions centered on RUNX3 binding sites. RUNX3 binding sites were classified into 642 sites around TSS and 2,492 distal to TSS (left). Enriched *de novo* motifs at the 642 RUNX3 binding site around TSS and the 2,492 distal regulatory regions are presented (right). **D,** Diagrams of H3K27ac HiChIP loops for prediction of RUNX3 target genes. Significant interactions were calculated (*P* < 0.05) in 10-kb (left) and 25-kb resolution (right). In 10-kb resolution, 2,301 loops between TSS's, 4,768 loops between TSS and distal region and 5,569 loops between distal regions were found using H3K27ac HiChIP data. Of them, 827 loops containing RUNX3 binding sites in distal region were identified. In 25-kb resolution, 6,734 loops between TSS's, 13,092 loops between TSS and distal region, 12,466 loops between distal regions were found using H3K27ac HiChIP data. Of them, 2,357 loops containing RUNX3 binding sites in distal region were identified. **E,** Rate of differentially expressed genes in among the RUNX3 target genes predicted by H3K27ac HiChIP. A total of 178 and 58 genes were significantly downregulated and upregulated, respectively, by RUNX3 KO. **F,** Enriched pathways obtained from MSIgDB Hallmark 2020 2 and Gene Ontology Biological Process, analyzing significantly repressed RUNX3 target genes in RUNX3 KO cells.

To understand how RUNX3 drives metastasis in HGC-27 cells, we compared the transcriptomic profiles of HGC-27_CONTROL and HGC-27_RUNX3KO cells. A total of 1,005 genes were downregulated after RUNX3 KO (Fig. 3A). Among the top ranked downregulated genes were cell surface adhesion receptor *CD44* and mesenchymal marker *vimentin* (also known as *VIM*; Fig. 3A). One of the most commonly studied markers for cancer stem cells, *CD44* has been strongly implicated in both tumor initiation and metastasis (26). Interestingly, repression of *CD44* is necessary for p53-mediated tumor suppression (27). *VIM* is an intermediate filament protein that maintains the structural integrity of the cell and plays roles in cell migration, motility, and adhesion. Importantly, *VIM* is known to be involved in metastasis in various cancer types (28). Other metastasis-associated genes that were significantly downregulated in HGC-27_RUNX3KO cells include *DPYSL3, SNTB1, WNT5A, SNAIL2, IGFBP3, RUNX2*, and *TGFB3*. *Dihydropyrimidinase-like-3 (DPYSL3)* has been reported to modulate mitosis, migration, and epithelial–mesenchymal transition (EMT) in breast cancer (29). *Beta-1 syntrophin (SNTB1)* is a scaffold protein that organizes signal transduction complexes. *SNTB1* has recently been shown to regulate colorectal cancer progression and stemness through the Wnt/β-catenin signaling pathway (30). *WNT Family Member 5A (WNT5A)*, a ligand that activates the noncanonical branch of the Wnt pathway, promotes cancer cell invasion and migration. *Insulin-like growth factor-binding protein-3 (IGFBP-3)* is a p53-inducible tumor suppressor gene that has proapoptotic function (31).

Pathway analysis of the significantly repressed genes in RUNX3 KO cells based on MSigDB Hallmark (2020) ranked extracellular matrix (ECM) organization at the top of the hierarchy (Fig. 3B). ECM proteins play key roles in metastatic cascade, where changes in the ECM affect adhesion, intravasation, and extravasation. Of note, the significantly repressed genes in RUNX3 KO cells also includes genes related to regulation of cell migration. GO analysis indicated that the significantly repressed genes in RUNX3 KO cells are enriched with genes involved in EMT (Fig. 3B). These results show that a considerable fraction of RUNX3 target genes are involved in metastasis.

### Mapping RUNX3 Genomic Occupancy in HGC-27 Cells

To determine whether RUNX3 directly regulates the pro-metastatic genes, we performed chromatin immunoprecipitation with RUNX3 antibody, followed by sequencing (ChIP-seq), in control (Cont) and HGC-27_RUNX3KO (KO) cells. We identified 642 RUNX3 binding sites around transcription start sites (TSS) and 2,492 RUNX3 binding sites at distal regulatory regions (Fig. 3C). There was no obvious difference in the signals for histone modifications H3K27ac (marks active sites), H3K4me3 (marks promoters), and H3K4me1 (marks enhancer regions) when control and RUNX3 KO cells were compared (Fig. 3C). RUNX3 KO was therefore not associated with large-scale changes in histone modifications or promoter and enhancer activities. Our findings confirm that RUNX3 functions mainly as a highly specialized master regulator of development, rather than a regulator of global transcription activity. Motif analysis of the RUNX3 binding sites revealed strong enrichment of RUNX consensus binding motifs around the TSS (Fig. 3C). By contrast, RUNX3 binding sites distal to the TSS contained—in addition to RUNX motifs—significant enrichment of binding motifs for lineage-determining transcription factors such as the homeobox protein CDX2, the Lim-homeodomain protein LHX2, and the HMG box Tcf3/4 (also known as Tcf7l1/2; Fig. 3C).

### H3K27ac HiChIP Interactions Predict RUNX3 Upregulation of Genes Involved in EMT

**FIGURE 4:**
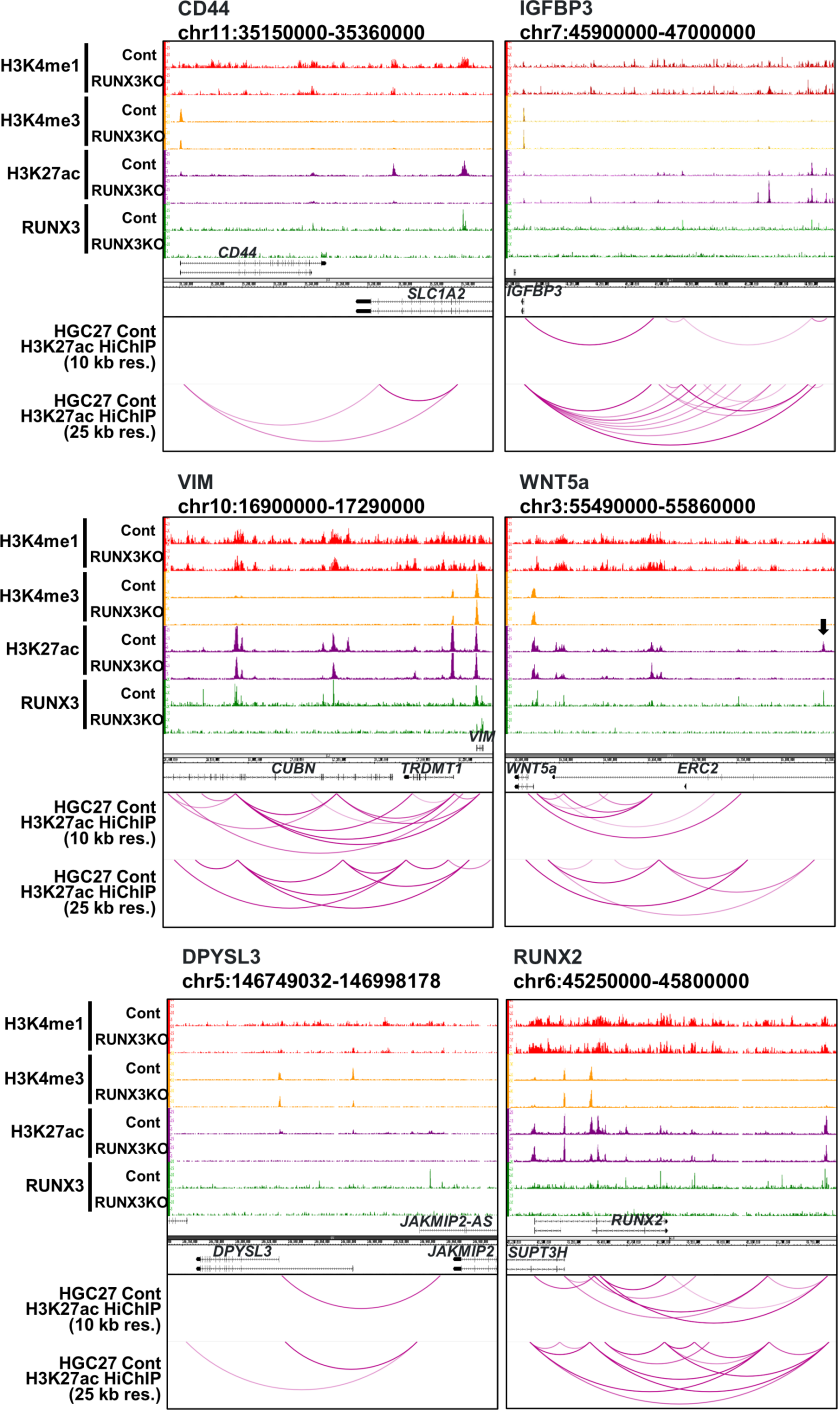
Histone modification and interactions proximal to genes transcriptionally activated by RUNX3. ChIP-seq analyses of histone modification and RUNX3 binding sites in HGC-27 control (cont) and RUNX3 KO cells are juxtaposed to H3K27ac HiChIP analyses. The representative genes include *CD44, IGFBP3, VIM, WNT5A, DPYSL3*, and *RUNX2*, based on the list of genes that showed high scores of *P* values and H3K27ac HiChIP profiles. Enhancer–promoter interactions are indicated by loops. Arrowhead indicates change in H3K27ac peak after RUNX3 KO.

RUNX3 distal binding sites may regulate gene expression via three-dimensional conformations of the chromatin, which bring them into spatial proximity with distant gene promoters. To identify loop structures between distal RUNX3 binding sites and their target promoters, we performed H3K27ac HiChIP. The entire genome was first divided into 10 kb segments and the interaction between each segment investigated through H3K27ac HiChIP loops (*P* < 0.05, 10 kb). There were 2,301 loops between TSS, 4,768 loops between TSS and distal regions and 5,569 loops between distal regions (Fig. 3D). Of them, there were 827 loops containing RUNX3 binding sites on the distal side. We next divided the entire genome into 25 kb segments for H3K27ac HiChIP analysis. There were 6,734 loops between TSS, 13,092 loops between TSS and distal parts, 12,466 loops between distal parts (Fig. 3D). Of these, there were 2,357 loops containing RUNX3 binding sites on the distal side. Using these interactions between distal RUNX3 binding sites and gene promoters, we identified 1,941 genes as distal RUNX3 targets. Of these, 178 of the RUNX3 target genes were significantly downregulated by RUNX3 KO, while 58 were significantly upregulated by RUNX3 KO (Fig. 3E). We next performed GO analysis (based on MSigDB Hallmark 2020) on the RUNX3 target genes which were downregulated by RUNX3 KO (*n* = 178). Analysis revealed EMT, hypoxia, and glycolysis as the top enriched pathways in the significantly repressed RUNX3 target genes in RUNX3 KO cells (Fig. 3F). Of note, *CD44, VIM, WNT5A*, and *IGFBP3* were predicted to be the EMT-related RUNX3 target genes (Fig. 3F). GO analysis (based on GO Biological Process) of the RUNX3 target genes which downregulated by RUNX3KO showed cell migration as the top enriched pathway in significantly repressed RUNX3 target genes in RUNX3 KO cells (Fig. 3F). In addition, we observed the involvement of RUNX3 in multiple developmental processes, such as ossification, limb morphogenesis, Wnt signaling, and kidney development (Fig. 3F). These data not only reflected the role of RUNX3 as master regulator of development, but also implicated RUNX3 as a driver of EMT during tumor metastasis. We selected *WNT5A, IGFBP3, CD44*, and *VIM* from the list of genes that showed high score of *P* value and HiChIP profiles. *VIM, CD44*, and *WNT5A* show significant interaction of their TSSs with distal RUNX3 regulatory regions (Fig. 4). In particular, the HiChIP interactions indicate that RUNX3 regulates *WNT5A* through binding to *WNT5A* promoter (Supplementary Fig. S4) and enhancer regions located in *ERC2* gene body (Fig. 4). While the H3K27ac peaks remained unchanged at the WNT5A promoter after RUNX3 KO, the H3K27ac peak was lost at the enhancer region in RUNX3 KO cells (see arrow in Fig. 4). Therefore, the ability of RUNX3 occupancy to influence H3K27ac modification is highly specific.

We next compared the HiChIP interactions with RUNX3 binding sites specifically enriched with motifs of CDX2, LHX2, and Tcf3 (also known as Tcf7l1; Fig. 3C). Analysis (based on MsigDB Hallmark 2020) of genes with active RUNX3 binding sites enriched with CDX2 motifs not only identified key EMT genes such as *CD44, SNTB1*, and *VIM*, but revealed EMT as one of the top five processes (Supplementary Data S1, page 1). Analysis of active RUNX3 binding sites with LHX2 motifs revealed EMT as the top ranked process, and includes EMT genes such as *DPYSL3, CD44, SNTB1*, and *VIM* (Supplementary Data S1, page 2). Finally, we analyzed active RUNX3 binding sites enriched with Tcf3 motifs and again found EMT as the top process (Supplementary Data S1, page 3). Not surprisingly, the Wnt/β-catenin signaling pathway was also identified as one of the top pathways, with genes such as *CD44, SNTB1, VIM*, and *HAPLN1* containing active RUNX3 bindings sites with Tcf3 motifs. RUNX3, together with Tcf factors, therefore directly regulates a specific subset of Wnt-responsive target genes. Remarkably, CD44 and VIM were again top hits. Taken together with the RNA-seq data, these findings suggest that RUNX3 regulates EMT activity through upregulation of genes such as *CD44* and *VIM*.

Our results indicate that RUNX3 is the critical driver of the metastatic phenotype in HGC-27 cells. We also provide a previously unrecognized view of the potential synergisms between RUNX3 and CDX2, LHX2 and TCF3 in promoting the transcription of genes involved in cancer stem cell regulation and metastasis. The findings underscore the importance of considering the expression of these collaborating genes when evaluating RUNX3 activity. Our study on the ability of RUNX3 to upregulate *CD44* is in progress and will be reported in due course. Here, we further explore the RUNX3-WNT5A association in metastasis.

### WNT5A Acts a Pivotal Role in RUNX3-mediated Metastasis in Gastric Cancer Cells

**FIGURE 5:**
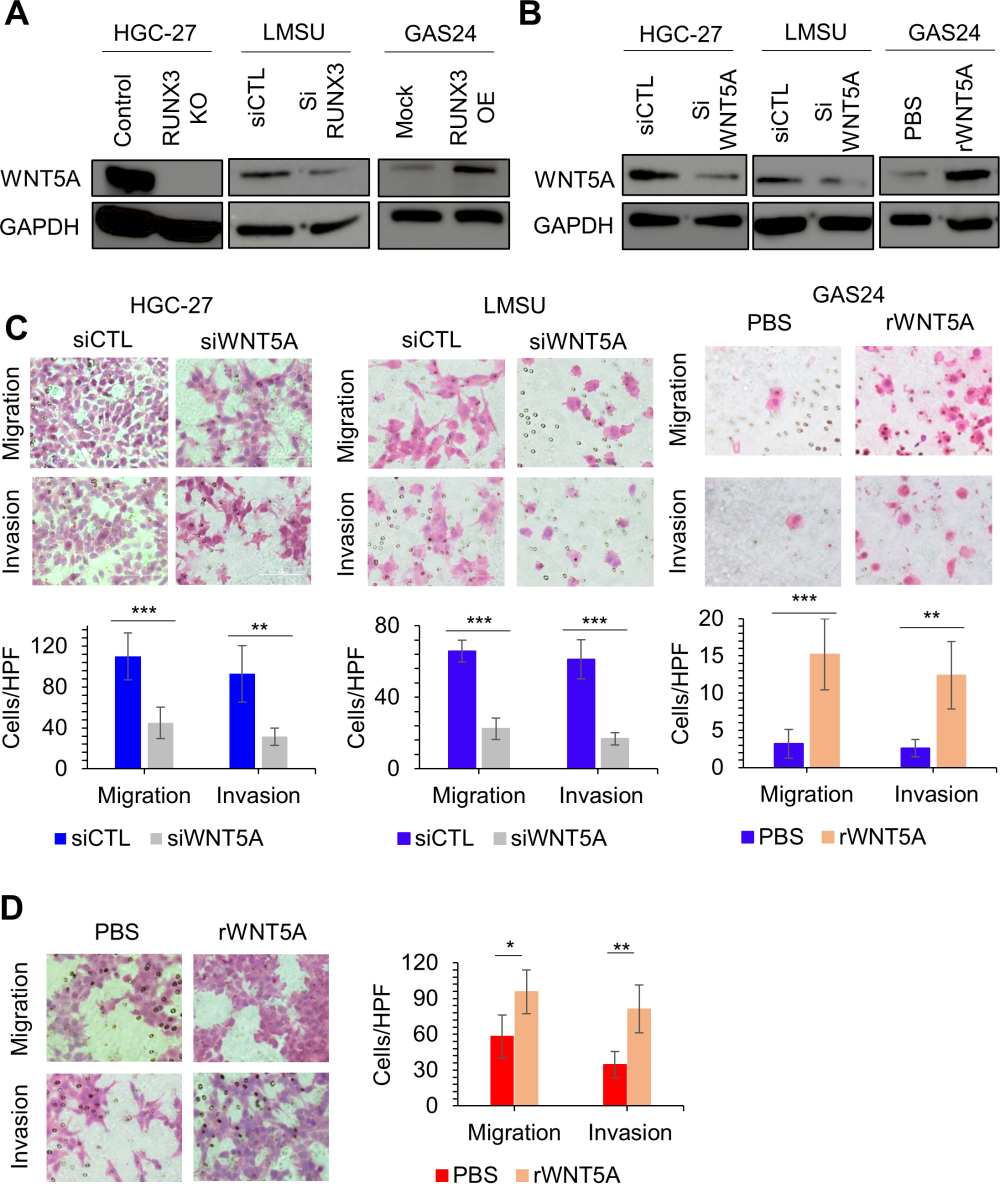
WNT5A plays a pivotal role in RUNX3-mediated metastasis in gastric cancer cells. **A,** immunoblot for WNT5A in HGC-27 after RUNX3 KO, LMSU after RUNX3 KD, and GAS24 after RUNX3 OE is shown. **B,** Immunoblot for WNT5A in HGC-27 and LMSU after siWNT5A, and in GAS24 after WNT5A recombinant treatment (0.1 mg/mL). **C,** Invasion and migration analysis for HGC-27 and LMSU after siRNA mediated WNT5A KD, and in GAS24 after WNT5A recombinant treatment (0.1 mg/mL). Experiments were repeated three times. Typical images from one experiment are shown (top). Cell invasion and migration were counted and quantified from 5 different high-power fields in each experiment (bottom); ***, *P* < 0.001 and **, *P* < 0.01 by a two-tailed Student *t* test. **D,** Invasion and migration analysis for HGC-27 KO and KO after WNT5A recombinant treatment (0.1 mg/mL). Experiments were repeated three times. Typical images from one experiment are shown (left). Cell invasion and migration were counted and quantified from 5 different high-power fields in each experiment (right); **, *P* < 0.01 and *, *P* < 0.05 by a two-tailed Student *t* test.

We investigated whether RUNX3 upregulates WNT5A in other gastric cancer cell lines. Similar to RUNX3 KO in HGC-27, siRNA-mediated RUNX3 KD in LMSU cells led to downregulation of WNT5A protein levels, whereas RUNX3 OE in GAS24 was correlated with increased WNT5A expression (Fig. 5A). To determine whether WNT5A is an important contributor of RUNX3-driven metastatic program in the stomach, we conducted siRNA-mediated KD of WNT5A in HGC-27 and LMSU. We also treated RUNX3-deficient GAS24 with recombinant WNT5A. WNT5A KD effectively downregulated WNT5A expression, while the WNT5A recombinant treatment upregulated WNT5A expression in GAS24 (Fig. 5B). WNT5A KD significantly decreased cell migration and invasion in HGC-27 and LMSU (Fig. 5C). The small decrease in proliferation after WNT5A KD was unlikely to impact migration/invasion during the 48 hours time frame of the assay (Supplementary Fig. S5A). Furthermore, recombinant WNT5A addition increased migration and invasion in GAS24 and RUNX3 KO HGC-27 cells (Fig. 5C and D; Supplementary Fig. S5B). Metastatic ability can also be assessed by anoikis resistance and anchorage independency, which allow detached tumor cells to expand and invade adjacent tissues (32). Anchorage-independent growth in Matrigel was inhibited by WNT5A KD in HGC-27 and LMSU cells, and promoted by WNT5A recombinant treatment in GAS24 (Supplementary Fig. S5C). We analysed TCGA database and found that patients with gastric cancer at stage IV expressed higher WNT5A mRNA expression compared with that at stage I (Supplementary Fig. S6), which indicates that WNT5A contributes to poorer prognosis and metastasis in patients with gastric cancer. Collectively, these results suggest that WNT5A plays critical roles in RUNX3-mediated metastasis.

**FIGURE 6:**
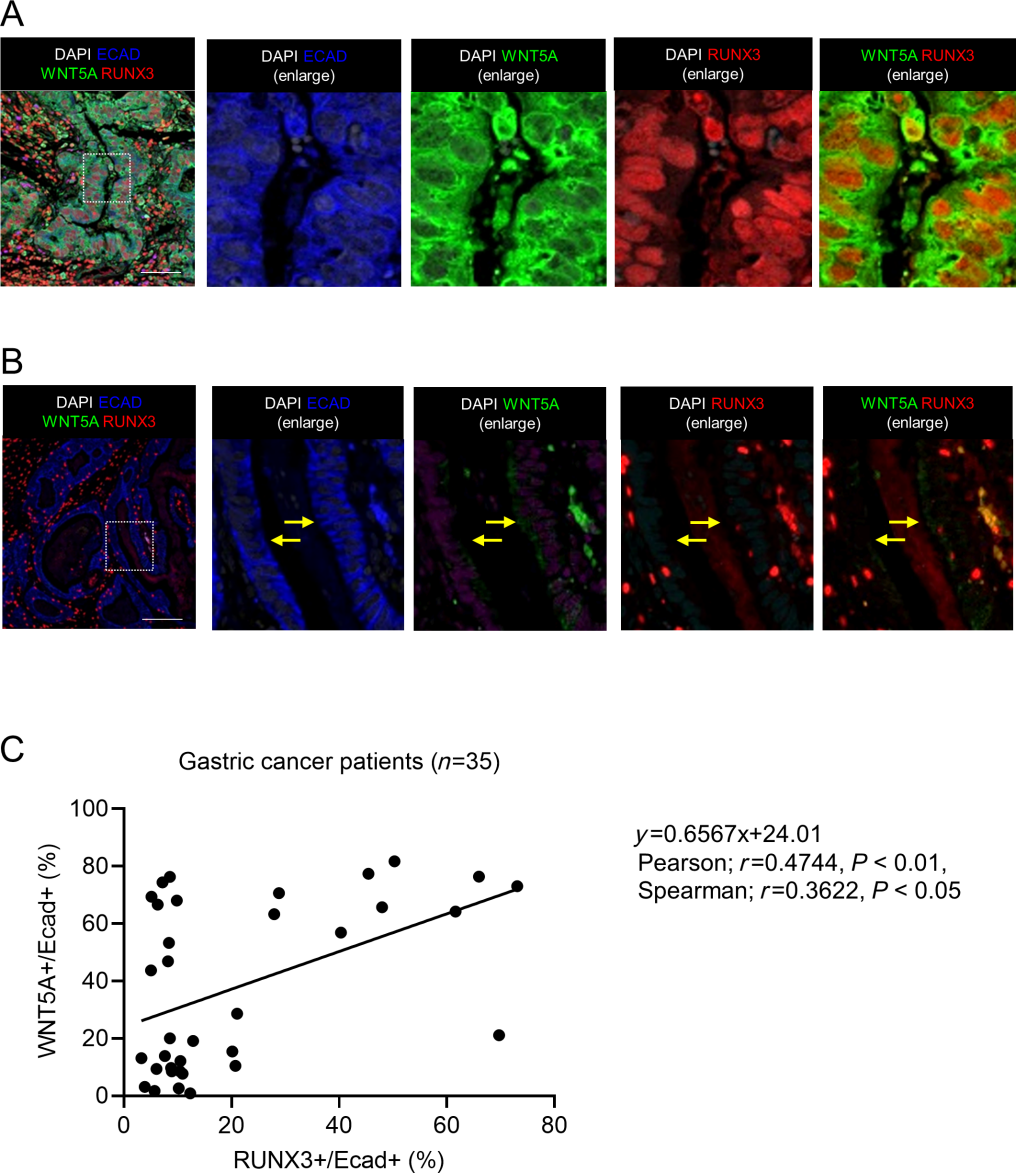
Direct WNT5A activation by RUNX3 would be a therapeutic strategy in gastric cancer. **A** and **B,** Representative imaging of immunofluorescence study in gastric cancer specimens (*n* = 35). A case showing high expression for RUNX3 (red) and WNT5A (green) in Ecadherin positive (blue) cancer cells (A) and a case showing negative RUNX3 and WNT5A expression in E-cadherin positive cancer cells (B) are shown. Arrows indicate the absence of RUNX3 and WNT5A in E-cadherin regions. Dotted boxes indicate enlarged regions. Scale bar: 50 µm. **C,** Positive correlation between RUNX3 and WNT5A expression in E-cadherin positive cancer cells by immunofluorescence study is indicated statistically (*n* = 35, Pearson; *r* = 0.4744, *P* < 0.01, Spearman; *r* = 0.3622, *P* < 0.05).

We next examine the coexpression of RUNX3 and WNT5A in E-cadherin positive gastric cancer cells in the human specimens (*n* = 35). Immunofluorescence staining revealed different expression patterns such as cases with coexpression of RUNX3 and WNT5A (Fig. 6A) as well as cases that lack both RUNX3 and WNT5A expression (Fig. 6B). The positive correlation between RUNX3 and WNT5A expression (Fig. 6C) could therefore be commonly observed in gastric cancer.

## Discussion

Here, we show that RUNX3 promotes cell migration, invasion, and anchorage-independent growth of gastric cancer cells *in vitro* and promotes tumor growth and metastasis *in vivo*. Immunofluorescence studies demonstrated stronger expression of RUNX3 in liver metastasized cells than the spleen tumors in splenic inoculation model, suggesting that RUNX3 confers a selective advantage during metastatic development. We found that RUNX3 promoted a transcriptional profile predominated by genes involved in developmental pathways that have been linked to the metastatic process. RUNX3 bound to regulatory regions of genes involved in EMT, survival, adhesion, angiogenesis, cell movement, and invasion. We show that RUNX3 directly upregulates metastasis-associated genes such as *VIM*, *IGFBP3, CD44*, and *WNT5A*. In particular, RUNX3 binds to the *WNT5A* gene and is required for strong *WNT5A* expression in the gastric cancer cell line HGC-27. Moreover, RUNX3-driven metastasis is mainly mediated by *WNT5A*, such that the depletion of WNT5A is sufficient to impair metastasis. Aberrant upregulation of developmental regulator RUNX3 may therefore be hijacked to drive metastasis in adult tissues.

Paradoxically, RUNX3 suppresses tumorigenesis in multiple tissue types. Mice with heterozygous deletion of *Runx3* induced adenoma in the lung, intestine, and mammary gland within a year, indicating that Runx3 is a gatekeeper for early stages of cancer (5). RUNX3 can suppress gastric epithelial cell growth by inducing cyclin-dependent kinase inhibitor *CDKN1A* expression (33). RUNX3 has the ability to induce premature senescence when expressed ectopically, suggesting that RUNX3 may induce senescence in response to oncogene-induced growth arrest (34). Not surprisingly, 60% of patients with gastric cancer do not express RUNX3 due to hemizygous deletion and hypermethylation of promoter region (14).

*RUNX3* expression was reported to be influenced by genes that are frequently mutated in cancer, namely *DPC4/SMAD4* and *p53* (13). Moreover, upregulation of *RUNX3* expression in response to DNA damage was observed previously (35). These observations indicate that RUNX3 is induced during oncogenic stress as a protective response against carcinogenesis. Here, we show that a large proportion of RUNX3-direct target genes is linked to tissue development and cell differentiation, such as bone development, ECM organization, cell adhesion, organ morphogenesis, chemotaxis, axon guidance, and neurogenesis. Moreover, we and others found that RUNX3 play a major role in the development of proprioceptive sensory neurons (36–39). In fact, *RUNX* genes were first discovered as developmental regulators through their development-specific regulation of tumor viral replication (40). When these developmental processes are engaged by the metastatic cascade, RUNX3 would inadvertently be an accomplice.

A key finding of our work is that RUNX3 regulates a large fraction of genes through binding to distal regions. Moreover, GO analysis of RUNX3-targeted enhancer regions revealed that WNT5A was ranked in the top 10 significantly enriched GO categories of biological processes. TCGA database revealed high WNT5A expression in patients with stage IV gastric cancer compared with stage IA. We demonstrated positive correlation between RUNX3 and WNT5A expression in resected cancer tissue. WNT5A is known to activate the noncanonical Wnt pathway, which could activate or inhibit the β-catenin pathway in a receptor context-dependent manner (41–44). WNT5A is also implicated in metastasis of multiple cancer types, such as gastric, lung, colorectal, and oral squamous carcinoma (41, 45–47). The report that anti-WNT5A antibody inhibited the cell migration of gastric cancer cells (47) further supports our findings. The involvement of WNT5A signaling in cancer stem cell self-renewal (44), which is potentially associated with tumor initiation and metastasis, suggests that RUNX3 may cooperate with WNT5A to regulate cancer stem property.

Whittle and colleagues reported that Runx3 regulates a notable number of genes implicated in ECM functions, including *Col6a1* and *Spp1*, to directly stimulate cell migration and dissemination and thereby promote metastasis in pancreatic cancer (13). However, we did not observe downregulation of *COL6A1* and *SPP1* in *RUNX3* KO of HGC-27. It is likely that RUNX3 promotes a distinct metastasis program in gastric cancer. Nevertheless, we observed that RUNX3 regulates a transcriptional profile heavily linked to ECM remodeling as well as other aspects of the metastatic cascade.

Our work revealed hitherto unknown interactions of RUNX3 with topologically associated domains in the neighborhoods of genes (e.g., *WNT5A, CD44, VIM*, and *IGFBP3*) with prominent roles in metastasis. These data provide mechanistic insights on how *RUNX3* may coordinate the metastatic program in gastric cancer. We also identified WNT5A as one of main effectors of RUNX3 in promoting metastasis. WNT5A is not only a good prognostic indicator but also a candidate for therapeutic target. A better understanding of RUNX3 regulation of *WNT5A* will yield insights to treatment of late-stage gastric cancer.

## Supplementary Material

Supplementary Data S1 The excel file shows active RUNX3 binding sites enriched with CDX2, LHX2 and TCF3 motifs and their corresponding GO biological processes at the Pages 1, 2 and 3, respectively.

Supplementary Figure S1 Higher RUNX3 expression in gastric cancer patients is associated with advanced stage and disease progression A, B, TCGA gastric cancer patient dataset visualized by XENA software (UCSC, n=591). Comparison of the mRNA expression of RUNX3 between normal tissues (n=38) and primary tumors (n=411) (A) as well as between stage Ia (n=17) and stage IV (n=49) (B) are shown; **, P < 0.01 and *, P < 0.05 by a two-tailed Student t test. C, TCGA gastric cancer patient dataset from microarray (cBioPortal) was obtained to determine RUNX3 mRNA expression in patients with disease-free (n=22) or with recurrence and disease progression (n=5); *, P < 0.05 by a two-tailed Student t test. D, comparison of survival data in RUNX3 high (red, n=349) or low (black, n=526) patients samples using microarrays obtained from KM-plotter database; P < 0.001.

Supplementary Figure S2 RUNX3 promotes cancer cell migration, invasion and anchorage independent growth in gastric cancer cells A, RT-qPCR analysis for RUNX3 mRNA in HGC-27 after RUNX3 KO; ****, P < 0.0001 by a twotailed Student t test. B, immunoblot shows absence of RUNX3 protein in HGC-27 by CRISPR/Cas9 mediated KO. C, WST-1 cell proliferation assay in HGC-27 after RUNX3 KO; **, P < 0.01 by a two-tailed Student t test. D, invasion and migration analysis for HGC-27 after RUNX3 KO. Experiments were repeated three times. Typical images from one experiment are shown. E, cell invasion and migration were counted and quantified from 5 different high-power fields in each experiment; ***, P < 0.001 by a two-tailed Student t test. F, immunoblot shows overexpression (OE) of RUNX3 protein in HGC-27 RUNX3 KO cells. G, invasion and migration analysis for HGC-27 RUNX3 KO cells after RUNX3 OE. Experiments were repeated three times. Typical images from one experiment are shown. H, cell invasion and migration were counted and quantified from 5 different high-power fields in each experiment; **, P < 0.01 and *, P < 0.05 by a two-tailed Student t test. I, representative images for HGC-27 after RUNX3 KO in soft agar colony formation assay. J, the number of colony formation was counted and shown as a fold change to control cells (mean + SD); **, P < 0.01 by a two-tailed Student t test.

Supplementary Figure S3 RUNX3 expression is higher in liver metastasis than in the primary tumor in splenic inoculation model A, RT-qPCR indicates relative RUNX3 expression obtained from tumors in spleen and liver metastasis after inoculation of HGC-27 control cells; **, P < 0.01 by a two-tailed Student t test. B, representative images of immunofluorescence study to compare RUNX3 expression obtained from tumors in spleen and liver metastasis after inoculation of HGC-27 control cells. Imaging for tumors of the spleen by RUNX3 KO cells is also shown as a negative control. Scale bar: 20 μm. C, quantitative analysis by image-J for fluorescence level of RUNX3 in spleen and liver metastasis after inoculation of HGC-27 control cells; **, P < 0.01 by a two-tailed Student t test.

Supplementary Figure S4 Chromatin status relative to RUNX3 peaks at the WNT5A promoter Enlarged image of the ChIPseq analysis of WNT5A locus in Fig. 4.

Supplementary Figure S5 Addition of exogenous recombinant WNT5A restores migration and invasion abilities to RUNX3 KO cells A, WST-1 cell proliferation assay in HGC-27 after siRNA mediated WNT5A KD; *, P < 0.05 by a two-tailed Student t test. B, immunoblot for WNT5A in HGC-27 KO cells after WNT5A recombinant treatment (0.1mg/mL) is shown. C, representative images for HGC-27 and LMSU after siWNT5A, and in GAS24 after WNT5A recombinant treatment (0.1mg/mL) in Matrigel colony formation assay (top). The number of colony formation was counted and shown as a fold change to control cells (mean + SD); **, P < 0.01 and *, P < 0.05 by a two-tailed Student t test.

Supplementary Figure S6 Higher WNT5A expression in gastric cancer patients is associated with advanced stage TCGA gastric cancer patient dataset was visualized by XENA software (UCSC, n=591). The RNA expression level of WNT5A between stage I (n=25) and stage IV (n=41) are shown; **, P < 0.01 by a two-tailed Student t test.
