# Supplementary figures and images for: Aberrant Upregulation of RUNX3 Activates Developmental Genes to Drive Metastasis in Gastric Cancer
Source: Cancer Res Commun. 2024 Feb 2;4(2):279–92. doi: 10.1158/2767-9764.CRC-22-0165 (PMC10836196; doi:10.1158/2767-9764.CRC-22-0165)

## Slide 1
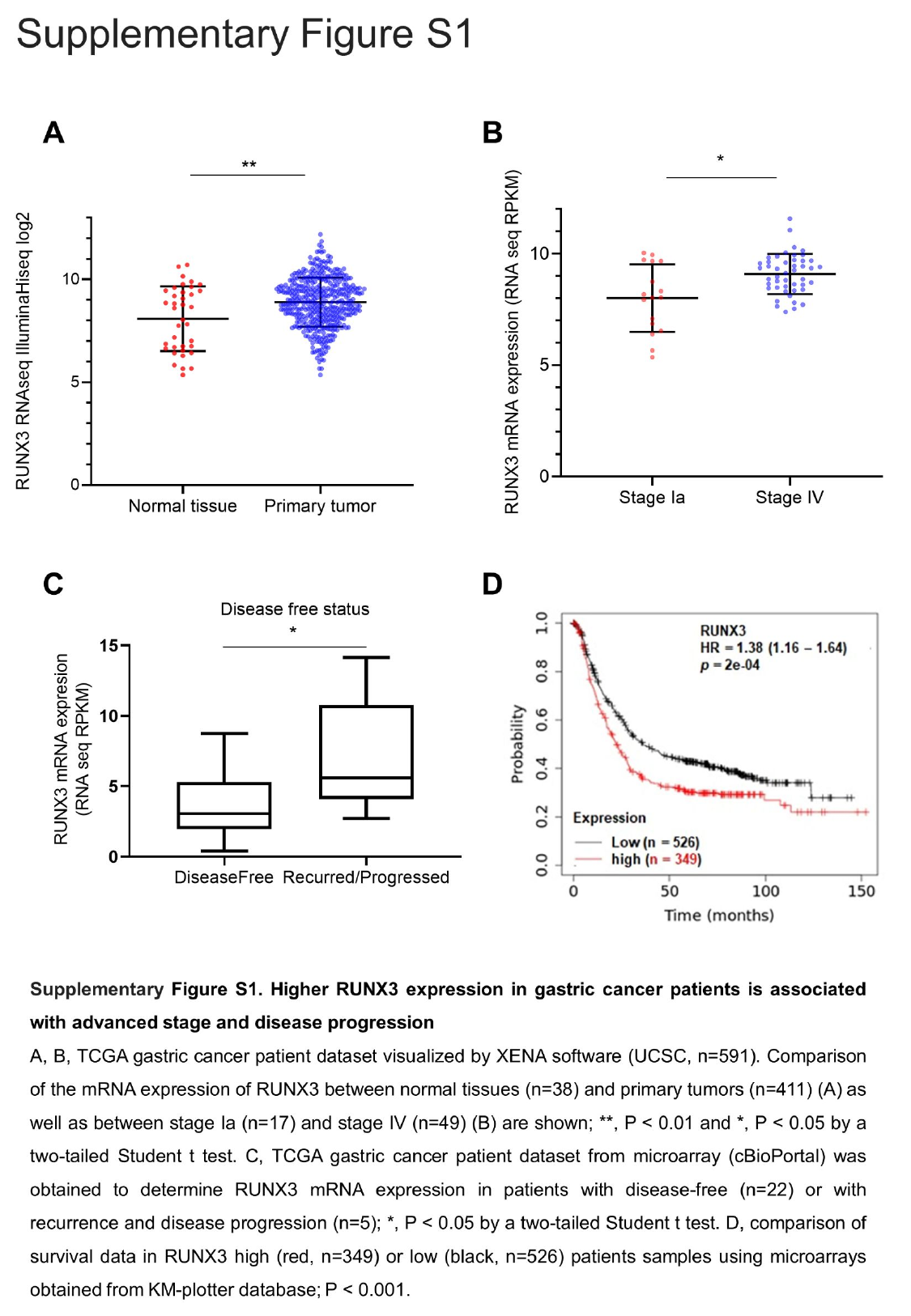

Supplement: Supplementary Figure S1 — Higher RUNX3 expression in gastric cancer patients is associated with advanced stage and disease progression A, B, TCGA gastric cancer patient dataset visualized by XENA software (UCSC, n=591). Comparison of the mRNA expression of RUNX3 between normal tissues (n=38) and primary tumors (n=411) (A) as well as between stage Ia (n=17) and stage IV (n=49) (B) are shown; **, P < 0.01 and *, P < 0.05 by a two-tailed Student t test. C, TCGA gastric cancer patient dataset from microarray (cBioPortal) was obtained to determine RUNX3 mRNA expression in patients with disease-free (n=22) or with recurrence and disease progression (n=5); *, P < 0.05 by a two-tailed Student t test. D, comparison of survival data in RUNX3 high (red, n=349) or low (black, n=526) patients samples using microarrays obtained from KM-plotter database; P < 0.001. [file crc-22-0165-s02.pptx]

## Slide 1
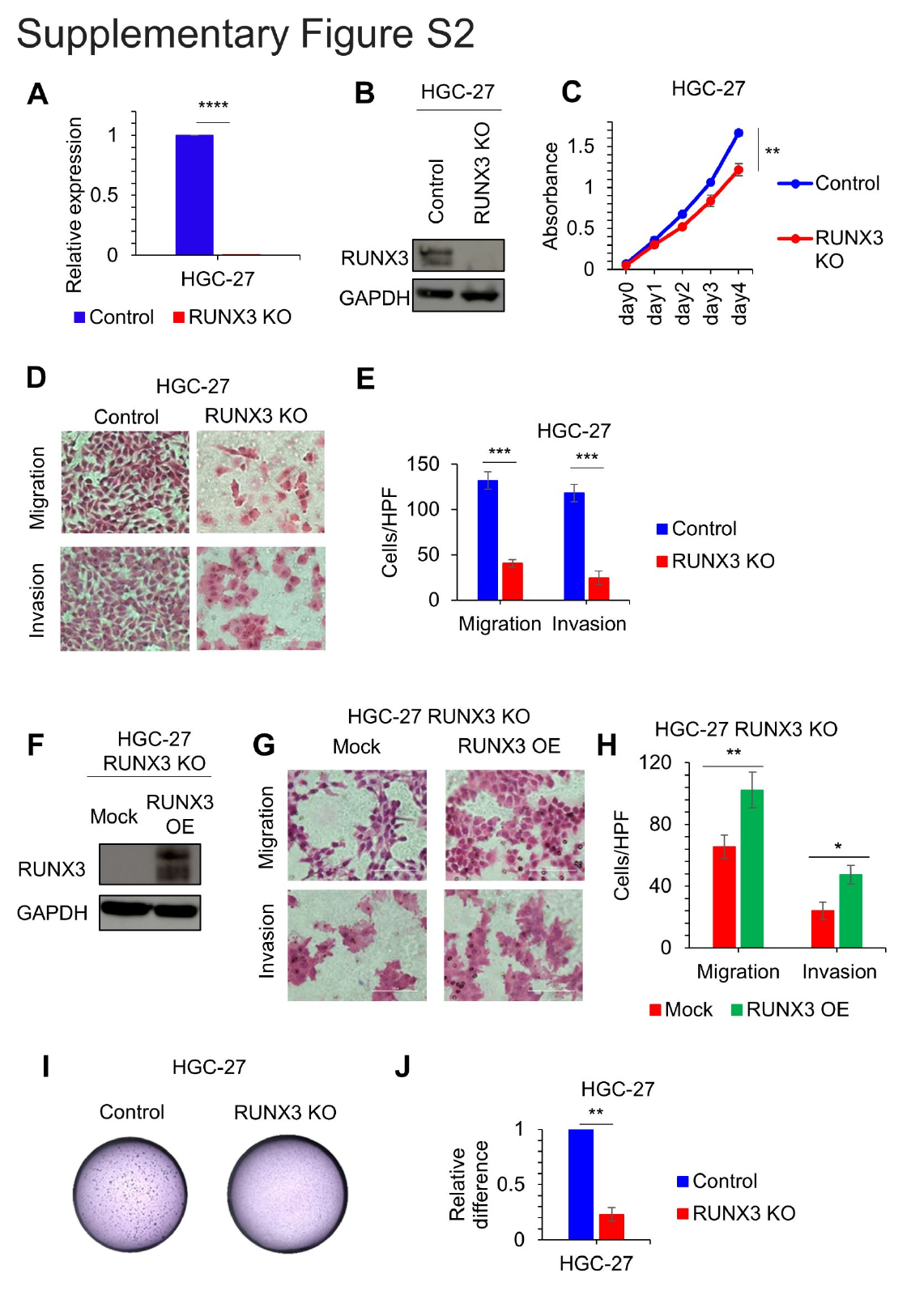

## Slide 2
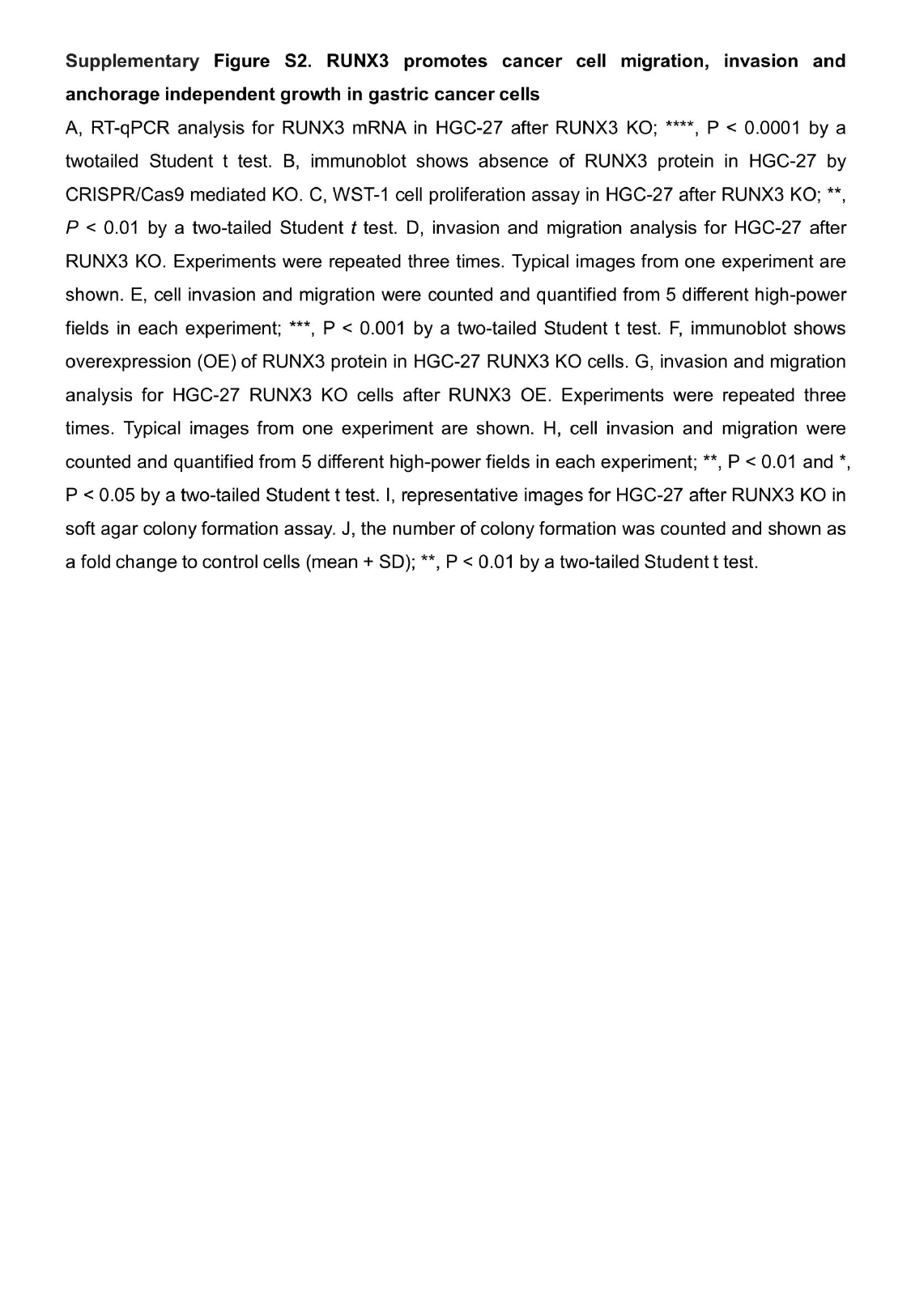

Supplement: Supplementary Figure S2 — RUNX3 promotes cancer cell migration, invasion and anchorage independent growth in gastric cancer cells A, RT-qPCR analysis for RUNX3 mRNA in HGC-27 after RUNX3 KO; ****, P < 0.0001 by a twotailed Student t test. B, immunoblot shows absence of RUNX3 protein in HGC-27 by CRISPR/Cas9 mediated KO. C, WST-1 cell proliferation assay in HGC-27 after RUNX3 KO; **, P < 0.01 by a two-tailed Student t test. D, invasion and migration analysis for HGC-27 after RUNX3 KO. Experiments were repeated three times. Typical images from one experiment are shown. E, cell invasion and migration were counted and quantified from 5 different high-power fields in each experiment; ***, P < 0.001 by a two-tailed Student t test. F, immunoblot shows overexpression (OE) of RUNX3 protein in HGC-27 RUNX3 KO cells. G, invasion and migration analysis for HGC-27 RUNX3 KO cells after RUNX3 OE. Experiments were repeated three times. Typical images from one experiment are shown. H, cell invasion and migration were counted and quantified from 5 different high-power fields in each experiment; **, P < 0.01 and *, P < 0.05 by a two-tailed Student t test. I, representative images for HGC-27 after RUNX3 KO in soft agar colony formation assay. J, the number of colony formation was counted and shown as a fold change to control cells (mean + SD); **, P < 0.01 by a two-tailed Student t test. [file crc-22-0165-s03.pptx]

## Slide 1
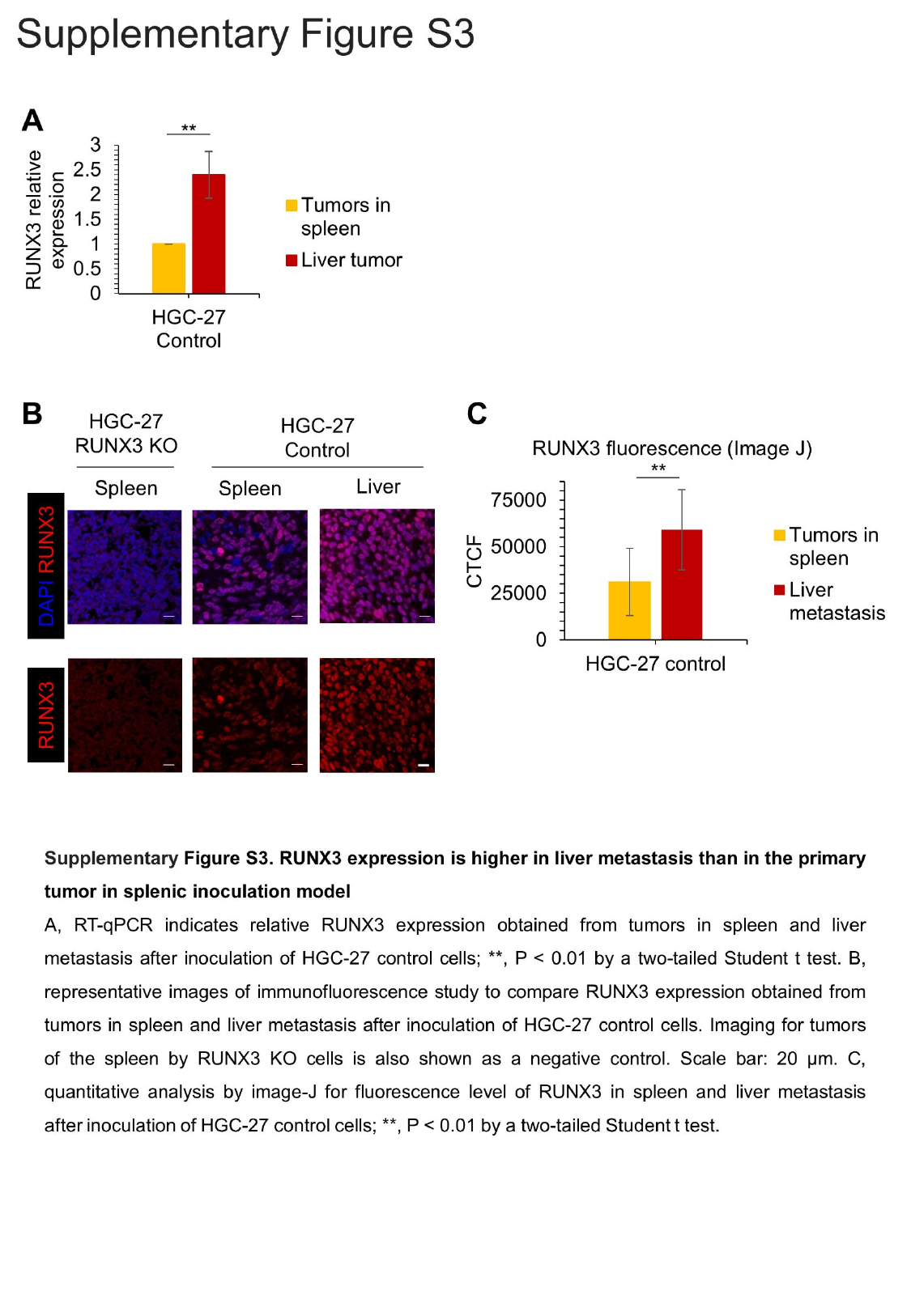

Supplement: Supplementary Figure S3 — RUNX3 expression is higher in liver metastasis than in the primary tumor in splenic inoculation model A, RT-qPCR indicates relative RUNX3 expression obtained from tumors in spleen and liver metastasis after inoculation of HGC-27 control cells; **, P < 0.01 by a two-tailed Student t test. B, representative images of immunofluorescence study to compare RUNX3 expression obtained from tumors in spleen and liver metastasis after inoculation of HGC-27 control cells. Imaging for tumors of the spleen by RUNX3 KO cells is also shown as a negative control. Scale bar: 20 μm. C, quantitative analysis by image-J for fluorescence level of RUNX3 in spleen and liver metastasis after inoculation of HGC-27 control cells; **, P < 0.01 by a two-tailed Student t test. [file crc-22-0165-s04.pptx]

## Slide 1
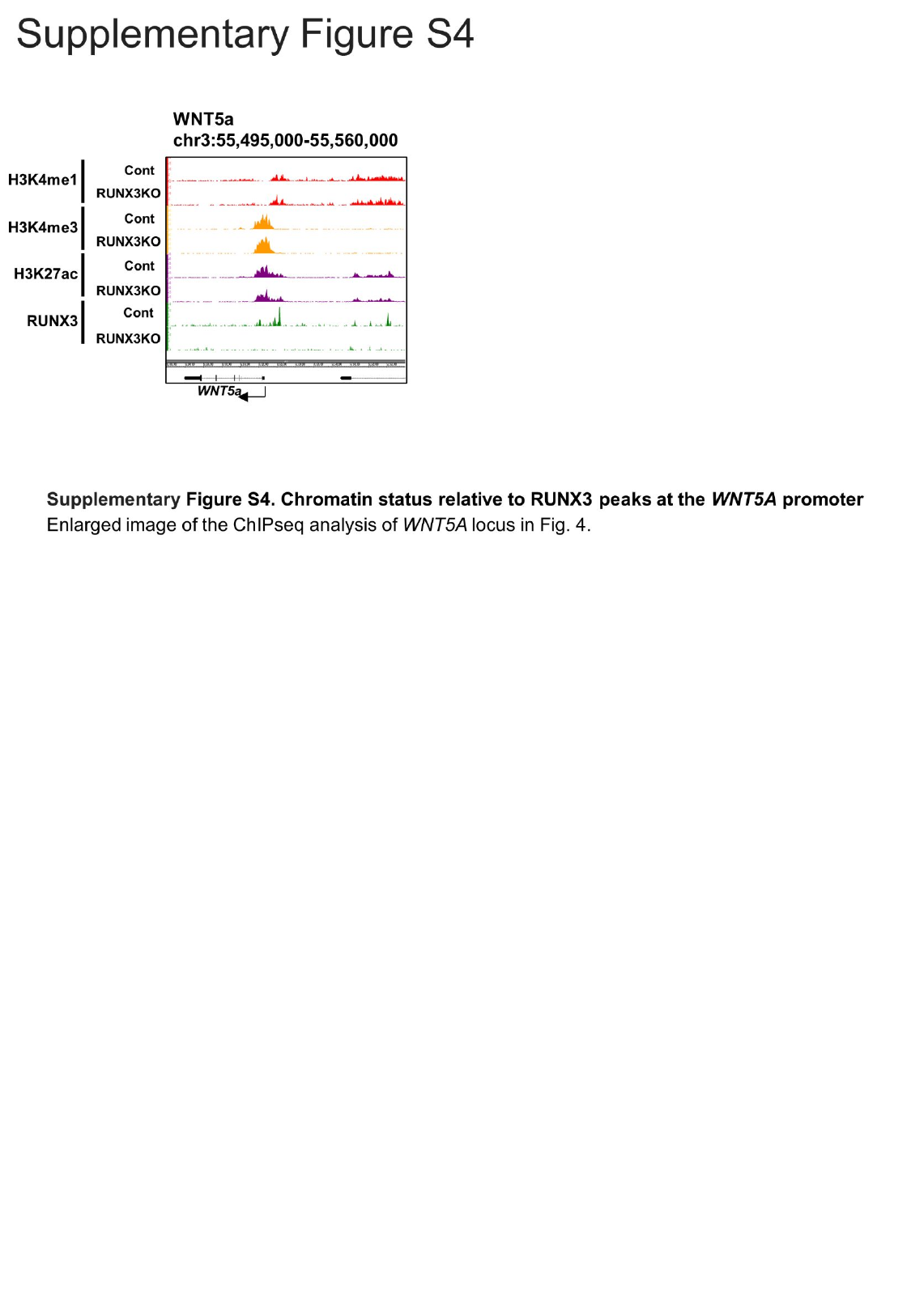

Supplement: Supplementary Figure S4 — Chromatin status relative to RUNX3 peaks at the WNT5A promoter Enlarged image of the ChIPseq analysis of WNT5A locus in Fig. 4. [file crc-22-0165-s05.pptx]

## Slide 1
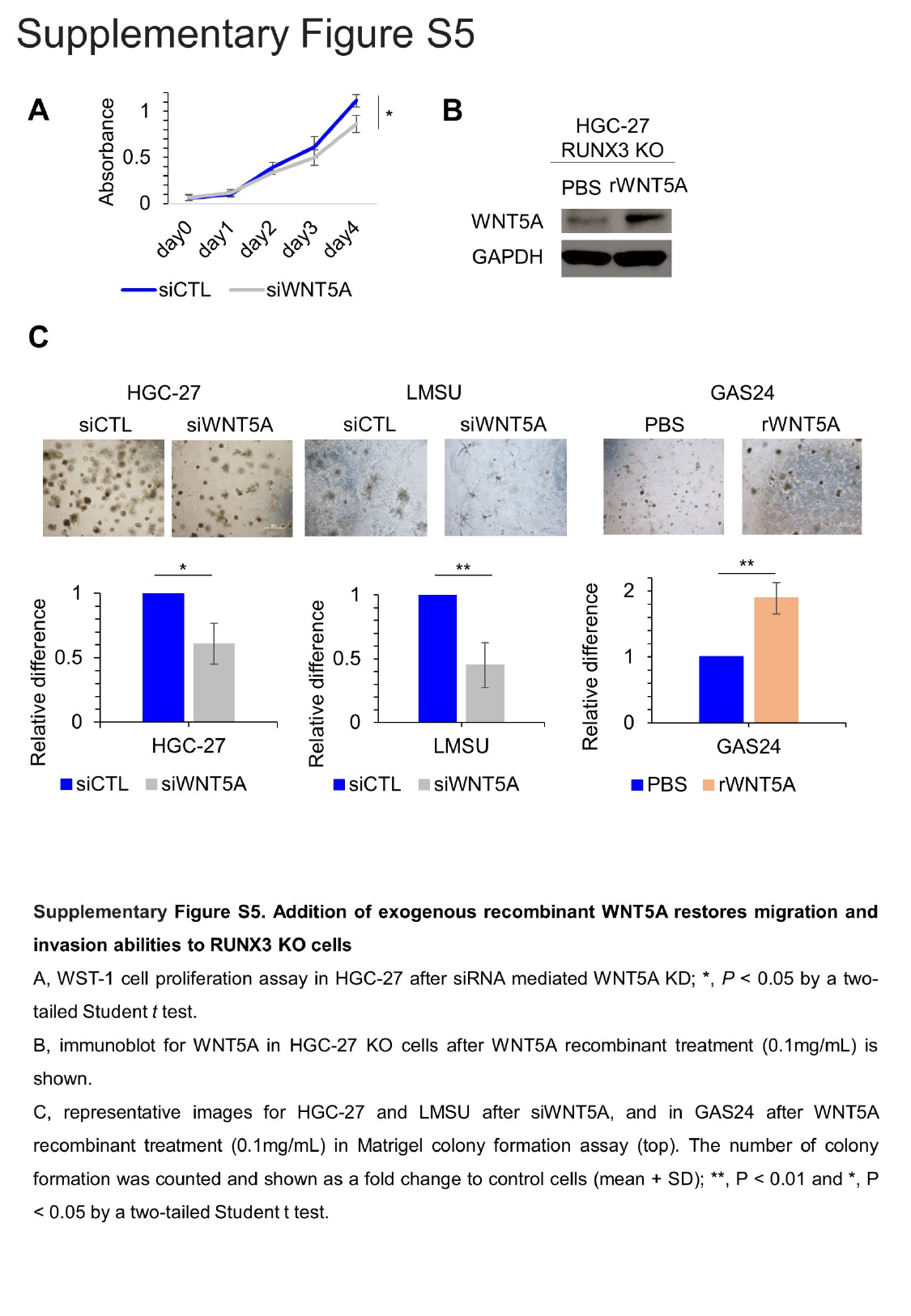

Supplement: Supplementary Figure S5 — Addition of exogenous recombinant WNT5A restores migration and invasion abilities to RUNX3 KO cells A, WST-1 cell proliferation assay in HGC-27 after siRNA mediated WNT5A KD; *, P < 0.05 by a two-tailed Student t test. B, immunoblot for WNT5A in HGC-27 KO cells after WNT5A recombinant treatment (0.1mg/mL) is shown. C, representative images for HGC-27 and LMSU after siWNT5A, and in GAS24 after WNT5A recombinant treatment (0.1mg/mL) in Matrigel colony formation assay (top). The number of colony formation was counted and shown as a fold change to control cells (mean + SD); **, P < 0.01 and *, P < 0.05 by a two-tailed Student t test. [file crc-22-0165-s06.pptx]

## Slide 1
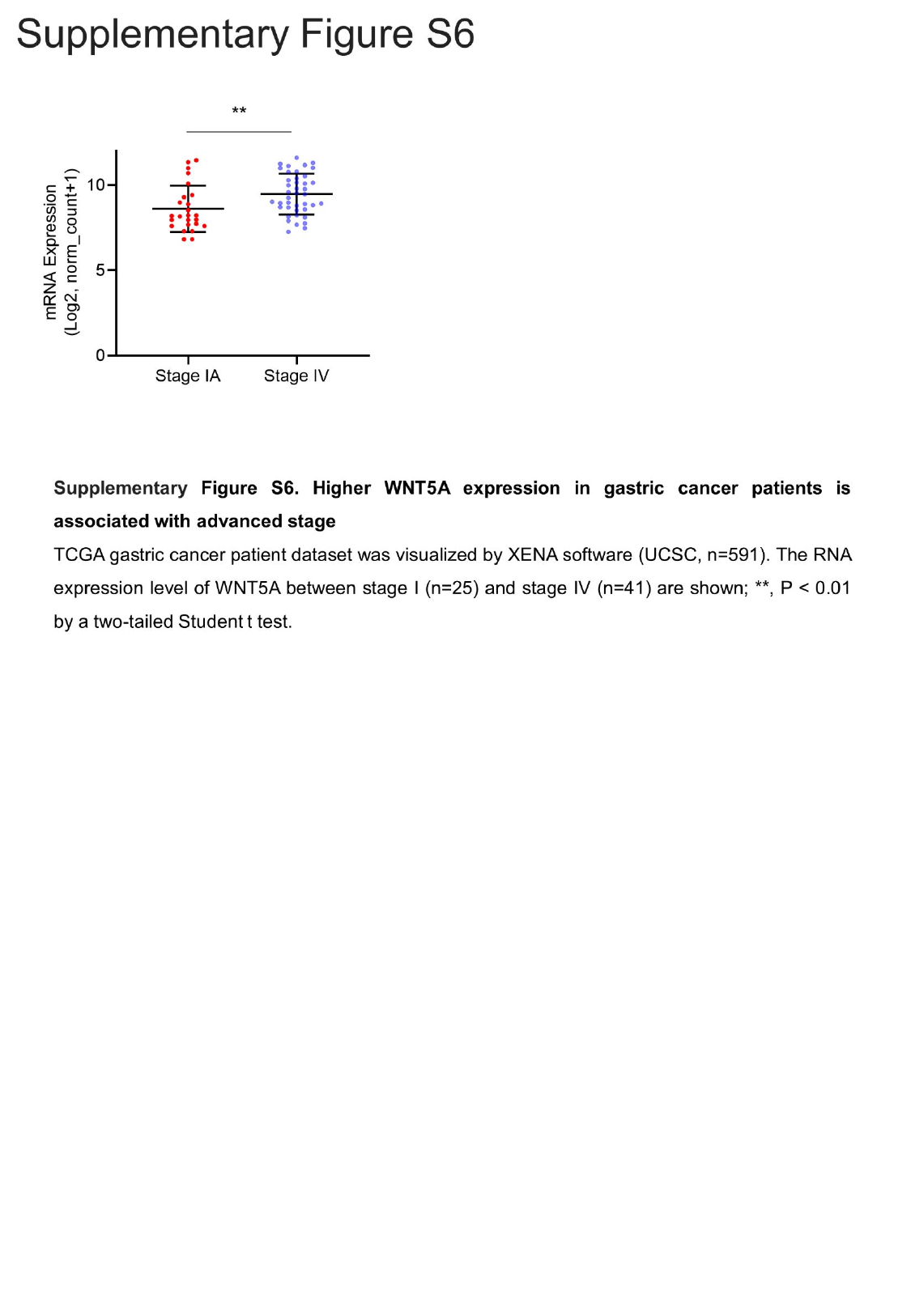

Supplement: Supplementary Figure S6 — Higher WNT5A expression in gastric cancer patients is associated with advanced stage TCGA gastric cancer patient dataset was visualized by XENA software (UCSC, n=591). The RNA expression level of WNT5A between stage I (n=25) and stage IV (n=41) are shown; **, P < 0.01 by a two-tailed Student t test. [file crc-22-0165-s07.pptx]
